# Analysis of in vivo turnover of tau in a mouse model of tauopathy

**DOI:** 10.1186/s13024-015-0052-5

**Published:** 2015-10-26

**Authors:** Kaoru Yamada, Tirth K. Patel, Katja Hochgräfe, Thomas E. Mahan, Hong Jiang, Floy R. Stewart, Eva-Maria Mandelkow, David M. Holtzman

**Affiliations:** Department of Neuropathology, Graduate School of Medicine, The University of Tokyo, Tokyo, 113-0033 Japan; Department of Neurology, Hope Center for Neurological Disorders, Knight Alzheimer’s Disease Research Center, Washington University School of Medicine, St. Louis, Missouri 63110 USA; MPI for Neurological Research, Hamburg Outstation, c/o DESY, Notkestr. 85, 22607 Hamburg, Germany; DZNE (German Ctr Neurodegen. Diseases), Ludwig-Erhard-Allee 2, 53175 Bonn, Germany; CAESAR Research Center, Ludwig-Erhard-Allee 2, 53175 Bonn, Germany

**Keywords:** Alzheimer’s disease, Clearance, Half-life, Extracellular tau, Tauopathy model

## Abstract

**Background:**

Intracellular accumulation of tau as neurofibrillary tangles (NFTs) is the hallmark of Alzheimer’s disease (AD) as well as in other tauopathies. Tau is present not only in the cytoplasm but also in the extracellular space such as cerebrospinal fluid (CSF) and brain interstitial fluid (ISF). Although clearance is one critical parameter leading to such intracellular/extracellular tau accumulation, in vivo turnover of tau has not been well characterized. The current study has attempted to precisely determine in vivo turnover rates of tau utilizing tet-off regulatable mice. In particular, we assessed intracellular tau and extracellular tau, soluble tau, insoluble tau and phosphorylated tau at certain sites utilizing a combination of in vivo microdialysis, biochemical analysis and specific ELISAs recognizing each species. To examine the effect of a tauopathy-associated mutation on tau clearance, half-lives of various tau species were compared between the mice with a FTDP-17 mutation that induces β-sheet formation, ΔK280 mutation (pro-aggregant mice) and control mice with additional β-sheet breaking mutations (anti-aggregant mice).

**Results:**

Here we report that tau is metabolized at much slower turnover rates in vivo than in cell culture. We found that insoluble tau in pro-aggregant mice had a significantly slower half-life (t_1/2_ = ~34.2 days) than soluble tau (t_1/2_ = ~9.7 days). In contrast, soluble tau phosphorylated in the proline rich region was cleared faster than total soluble tau. When comparing pro-aggregant mice to anti-agregant mice, turnover rates of soluble tau species were not significantly different.

**Conclusions:**

The current study provides a comprehensive description of in vivo turnover of various tau species present in mice that express human tau. The turnover rate of soluble tau was not significantly altered between pro-aggregant mice and anti-aggregant mice. This suggests that altered conformation by ΔK280 does not have a major impact on clearance pathways for soluble tau. In contrast, different tau species displayed different half-lives. Turnover was significantly delayed for insoluble tau whereas it was accelerated for soluble tau phosphorylated in the proline rich region. These differences in susceptibilities to clearance suggest that aggregation and phosphorylation influences tau clearance which may be important in tau pathogenesis.

## Background

Abnormal tau aggregates as NFTs are common pathological hallmarks in a set of neurodegenerative diseases called tauopathies including AD, progressive supranuclear palsy, corticobasal degeneration, and certain forms of frontotemporal lobar dementia. Tau is physiologically highly soluble, however under pathological conditions, it changes its conformation to one with a high β–sheet content, undergoes hyperphosphorylation and forms aggregates including filaments and NFTs in the cytoplasm.

NFTs are formed intracellularly, however, extracellular tau also has pathological significance. For example, tau in CSF is elevated in individuals with AD and preclinical AD. In addition, recent studies have demonstrated that tau pathology may be transmitted from neuron to neuron via the extracellular space [1–5].

Growing evidence suggests that tau contributes to neuronal dysfunction. Chronic inhibition of tau expression in various mouse models protects them against cognitive impairments [6–8]. This leads to the idea that lowering tau production may be a potential therapeutic strategy. Since clearance pathways mediate tau protein levels, the development of tau-lowering therapy would benefit from a better understanding of in vivo turnover of tau. While a number of studies have focused on how tau is metabolized in cultured cells [9–14], surprisingly little is known about in vivo clearance of tau, especially pathologically relevant tau species that accumulate in the tauopathies such as aggregated forms of tau, extracellular tau, and phosphorylated tau. Therefore, an analysis of the in vivo turnover rate of tau in mice that develop tauopathy will not only deepen our understanding of tau clearance at the systemic level but also assist in interpreting tau changes in the clinical setting.

In order to directly address this issue, we set out to evaluate in vivo turnover rates of tau in animal models that accumulate pathogenic tau species. In this study, we utilized tet-off tau transgenic mice called pro-aggregant mice, which express a regulatable human 2N4R tau cDNA with a ΔK280 tau mutation. These mice develop age-dependent tau aggregation [6, 15, 16]. We examined turnover rates of intracellular soluble tau, insoluble tau, phosphorylated tau and extracellular soluble tau by coupling biochemical analysis and in vivo microdialysis with specific ELISAs recognizing different tau species. Here we report that in vivo turnover rates of tau were significantly longer than in cell culture. Susceptibility to clearance varies among different tau species. Intracellular insoluble tau displayed a significantly longer half-life in pro-aggregant mice and soluble tau phosphorylated in the proline rich region showed faster turnover rates than total soluble tau. When turnover rates of soluble tau were compared between pro-aggregant mice and aged matched control mice with a β-sheet breaking mutation in tau called anti-aggregant mice [17], significant differences were not observed.

## Results and discussion

### Strategy to investigate turnover of intracellular soluble tau, insoluble tau, phosphorylated tau and extracellular tau in tet-off regulatable tau transgenic mice

**Fig. 1:**
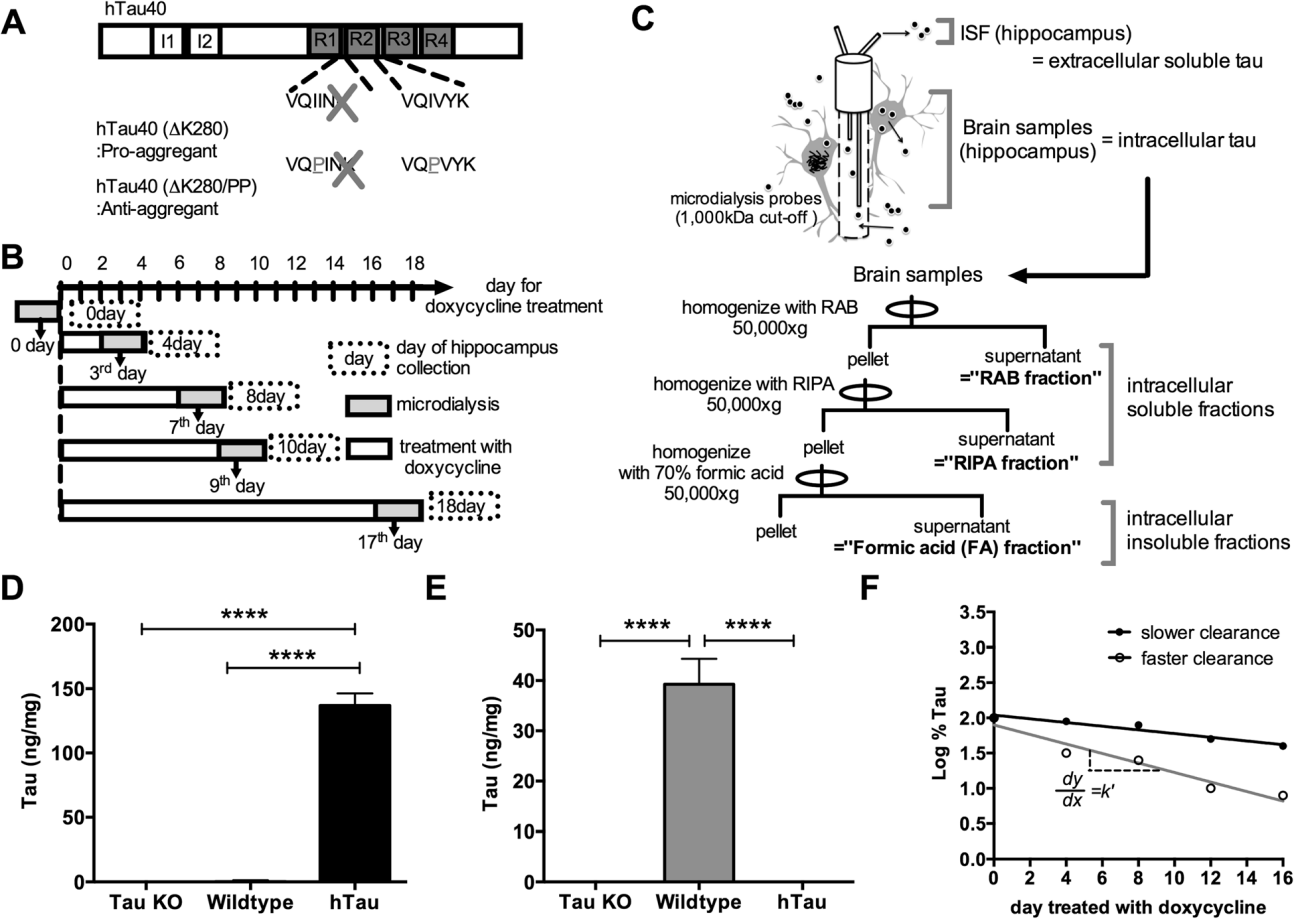
Schematic illustration of regulatable tau transgenic mice and experimental design. **a** Illustration of tau transgene in pro-aggregant mice and anti-aggregant mice. **b** A schematic representation of in vivo microdialysis during doxycycline treatment. Human tau expression was suppressed by doxycycline in 16–17 month old pro-aggregant mice or anti-aggregant mice for a period indicated in the white box. ISF was collected for 2 days indicated in the gray box while doxycyline administration continued. The mean levels of ISF human tau were plotted against time indicated in arrows. Immediately after microdialysis experiments, the hippocampus was dissected (day of hippocampus collection). **c** Experimental scheme for collection of extracellular and intracellular tau in this study. Microdialysis probes with 1,000 kDa cut-off membranes were inserted in hippocampus to collect soluble extracellular tau in ISF. To measure intracellular tau, the hippocampus was homogenized in 3-step serial extraction with RAB, RIPA and 70 % Formic acid (FA) (See the details in methods section). **d** Tau5/HT7B ELISA specifically detects human tau. Tau levels in RAB fractions from Tau knockout mice (Tau KO) or wildtype mice or hTau mice were analyzed by Tau5/HT7B ELISA (*n* = 4/group, ****p < 0.0001). **e** HJ9.2/HJ8.7B ELISA specifically detects murine tau. Tau levels in RAB lysates from Tau knockout mice (Tau KO) or wildtype mice or hTau mice were analyzed by HJ9.2/HJ8.7B ELISA (*n* = 4/group, ****p < 0.0001). **f** Schematic diagram for calculation of half-life. Log-transformed values are fit with a linear regression and slope k’ was obtained from linear regression to calculate t_1/2_. Differences in slopes reflect alteration in clearance (white circles for faster clearance, black circles for slower clearance)

To examine turnover rates of various tau species in vivo, we utilized tet-off tau transgenic mice, called pro-aggregant mice [6, 15, 16]. Pro-aggregant mice express a 2N4R isoform of human tau with a ΔK280 mutation, which is found in a family with FTDP-17 [18] (Fig. 1a). This mutation modifies MAPT splicing leading to an increase of three repeat isoform, however four repeat isoform with this ΔK280 mutation is still produced [18, 19], which enhances propensity of tau to form β-sheet structure. In these mice, tau changes its conformation to a form containing high β-sheet content, forms insoluble aggregates and undergoes hyperphosphorylation, which allows us to assess various pathologically relevant tau species. Although the 16–17 month old mice that we utilized in this study had not yet developed pronounced NFTs at this age, they have detergent insoluble tau [16] and display synapse loss and cognitive decline, which can be reversed when tau expression is suppressed [6].

The expression of human tau in these mice can be specifically switched off by doxycycline. Therefore the rate at which tau steady-state levels decline following doxycycline treatment will reflect its half-life. In addition, the expression of human tau is kept intentionally low to avoid disturbance of endogenous metabolism in these mice.

The outline of our experimental design is illustrated in Fig. 1. Tau is present not only in the intracellular compartment but also in the extracellular space. To distinguish clearance of extracellular tau from intracellular tau, microdialysis was performed in the hippocampus during doxycycline treatment prior to brain collection (Fig. 1b, c). Since the microdialysis technique works on the principle of the diffusion of molecules in solution across a semi-permeable membrane, microdialysis samples contain extracellular soluble tau in ISF which is less than 1,000 kDa, the molecular weight cut-off of the probes [20].

To examine intracellular tau, hippocampal homogenates were prepared at the end of doxycycline treatment (Fig. 1b, c). Brain samples include both intracellular and extracellular proteins. However tau is predominantly present intracellularly [21], thus tau in hippocampal homogenates represents mostly intracellular tau (Fig. 1c). The hippocampus was immediately dissected after microdialysis experiments and biochemically processed by serial extraction using reassembly buffer (RAB), RIPA, 70 % formic acid to obtain soluble and insoluble intracellular fractions (Fig. 1b, c). Tau levels were quantified in these fractions (We defined each fraction as follows; ISF = extracellular soluble fraction, hippocampus soluble fraction = intracellular soluble fraction, hippocampus insoluble fraction = intracellular insoluble fraction) by ELISA (Fig. 1c). We utilized a combination of Tau5 and biotinylated HT7 (HT7B) antibodies to detect human tau and HJ9.2 and biotinylated HJ8.7 (HJ8.7B) antibodies to detect murine tau respectively. We first checked the specificity of these ELISAs using brain homogenates from hTau mice, which express human tau on a murine tau knockout background along with wildtype and tau knockout mice as controls [22]. Tau5/HT7B ELISA detected tau only in brain homogenates of hTau mice (Fig. 1d). In contrast, the HJ9.2/HJ8.7B ELISA only detected tau in wildtype mice (Fig. 1e). This demonstates that our assays are highly specific to human or murine tau. After tau levels were measured by ELISA, log-transformed tau levels were plotted over days treated with doxycycline along with a linear regression curve and turnover rates were determined by analyzing the slope as previously described [20, 23] (Fig. 1f).

### Turnover of soluble tau in pro-aggregant mice and anti-aggregant mice

**Fig. 2:**
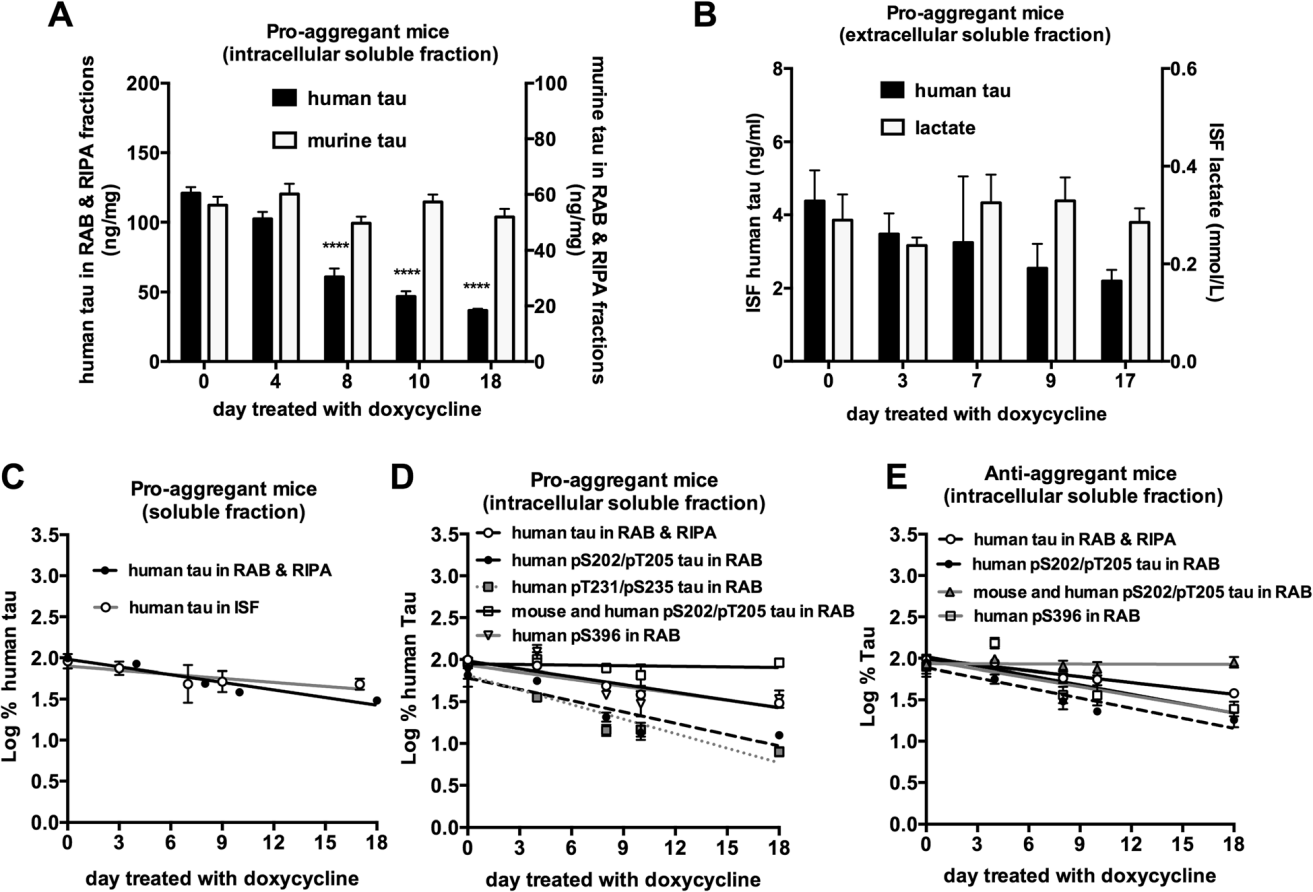
Turnover of soluble tau in pro-aggregant mice and anti-aggregant mice. **a** Doxycycline specifically reduced human tau in intracellular fractions of pro-aggregant mice. Human (black bars) and murine tau levels (white bars) in intracellular soluble fractions following doxycycline treatment were measured (*n* = 4 for day 10, *n* = 5 for day 4 and day 18, *n* = 7 for day 8, *n* = 12 for day 0. ****p < 0.0001) in pro-aggregant mice. **b** Doxycycline specifically reduced human tau in ISF of pro-aggregant mice. The levels of human tau (black bars) and lactate (white bars) in ISF of pro-aggregant following doxycycline treatment were measured (*n* = 6 for day 0, *n* = 5 for day 3, *n* = 4 for day 7, *n* = 4 for day 9, *n* = 5 for day 17, *n* = 4-6/group). **c** Semi-log plot of intracellular (black circles) and extracellular soluble human tau (white circles) changes (Log % soluble human tau) over time in pro-aggregant mice **d** Phosphorylated tau showed different clearance kinetics in brain of pro-aggregant mice. Semi-log plot of soluble human tau (white circles, Log % soluble human tau), and phosphorylated soluble tau (black circles, Log % soluble human pS202/pT205, gray squares, Log % soluble pT231/pS235 tau, white squares, Log % soluble murine and human pS202/pT205, white triangles, Log % soluble human pS396) over time in pro-aggregant mice. **e** Phosphorylated tau showed different clearance kinetics in the brain of anti-aggregant mice. Semi-log plot of soluble human tau (white circles, Log % soluble human tau), and phosphorylated soluble tau (black circles, Log % soluble human pS202/pT205, gray triangles, Log % phosphorylated murine and human tau pS202/pT205, white squares, Log % soluble human pS396) over time in anti-aggregant mice

By using hippocampal soluble fractions, we first investigated turnover rates of intracellular soluble tau in 16–17 month old pro-aggregant mice. In these fractions, doxycycline specifically reduced human but not murine tau as expected (Fig. 2a). A linear regression of semi-log plot (Fig. 2c) revealed that the estimated half-life of intracellular soluble tau in pro-aggregant mice was 9.7 days (Table 1). The half-life of tau determined in cell culture ranges from ~5 to 60 h [9–12, 24]. The significantly longer half-life of tau indicates that tau metabolism is different between cells and the in vivo environment.

**Table 1:** Half-lives of various tau species in pro-aggregant mice and anti-aggregant mice

Pro-aggregant mice		
	half-life (t_1/2_, day)	R^2^
Intracellular soluble	9.7	0.91
Extracellular soluble	17.3	0.7
* P*		0.06
intracellular soluble	9.7	0.91
intracellular soluble pS202/pT205	5.7	0.8
* P*		0.12
intracellular soluble	9.7	0.91
intracellular soluble pT231/pS235	5.2	0.88
* P*		<0.0001
intracellular soluble	9.7	0.91
intracellular insoluble	34.2	0.43
* P*		<0.0001
intracellular soluble	9.7	0.91
intracellular soluble pS396	9.8	0.51
* P*		0.52
Anti-aggregant mice		
	half-life (t_1/2,_ day)	R^2^
Intracellular soluble	11.1	0.96
Extracellular soluble	10.9	0.91
* P*		0.44
	half-life (t_1/2_, day)	R^2^
intracellular soluble	11.1	0.96
intracellular soluble pS202/pT205	7.5	0.89
* P*		0.022
	half-life (t_1/2_, day)	R^2^
intracellular soluble	11.1	0.96
intracellular soluble pS396	7.6	0.64
* P*		0.24

To examine clearance of extracellular tau, in vivo microdialysis was performed in the pro-aggregant mice. Doxycycline treated resulted in a trend of reduction in human tau with no change in ISF lactate (Fig. 2b). The half-life of extracellular tau in pro-aggregant mice was 17.3 days (Table 1). Although a trend was seen suggesting that extracellular soluble tau has a longer half-life than intracellular soluble tau (Table 1, intracellular soluble vs. extracellular soluble, *p* = 0.06), turnover rates were not significantly different (Fig. 2c).

Prior studies have linked dysfunction in protein metabolism with disease-associated mutations. For example, TDP-43 mutations linked to amyotrophic lateral sclerosis exhibit a longer half-life [25]. Alpha-synuclein with an A53T mutation associated with familial Parkinson’s disease also has increased stability [26]. It is unknown whether tau mutations could also have a similar effect on metabolism. In order to ask whether a longer half-life of tau in pro-aggregant mice is due to the conformational change driven by the ΔK280 mutation, turnover of tau in pro-aggregant mice was compared with 16–17 month old anti-aggregant mice where tau cannot form a β-sheet structure due to additional double proline mutations. There was no significant difference in half-lives of both intracellular soluble tau and extracellular soluble tau between pro-aggregant mice and anti-aggregant mice (Table 2). This suggests that altered conformation by the ΔK280 mutation does not have a profound effect on clearance of soluble tau. Importantly, in vivo half-lives of intracellular soluble tau determined for pro-aggregant mice and anti-aggregant mice are comparable to the half-life of endogenous murine tau in brain estimated by stable-isotope labeling [27]. This supports the idea that longer half-life of tau in tet-off tau transgenic mice is unlikely to derive from technical differences or over-expression.

**Table 2:** The comparison of half-life of tau between pro-aggregant mice and anti-aggregant mice

Intracellular soluble tau		
	half-life (t_1/2_, day)	R^2^
Pro-aggregant	9.7	0.91
Anti-aggregant	11.1	0.96
* P*		0.06
Extracellular soluble tau		
	half-life (t_1/2_, day)	R^2^
Pro-aggregant	17.3	0.7
Anti-aggregant	10.9	0.91
* P*		0.25
Intracellular soluble pS202/pT205 tau		
	half-life (t_1/2_, day)	R^2^
Pro-aggregant	5.7	0.8
Anti-aggregant	7.5	0.89
* P*		0.64
Intracellular soluble pS396 tau		
	half-life (t_1/2_, day)	R^2^
Pro-aggregant	9.8	0.51
Anti-aggregant	7.6	0.64
* P*		0.45

Aberrant phosphorylation of tau is one of the key features of AD. To see whether phosphorylation alters turnover of soluble tau, we investigated the half-life of human phosphorylated tau at S202/T205 and T231/S235 by using human tau specific HJ8.5 antibody [28] along with phospho-specific antibodies AT8 and AT180 antibodies in ELISAs. We found that intracellular soluble tau phosphorylated at S202/T205 and T231/S235 displayed faster turnover rates than total soluble tau in pro-aggregant mice (Fig. 2d). The half-life of tau phosphorylated at S202/T205 and T231/S235 was 5.7 days and 5.2 days respectively (Table 1). The turnover rate of phosphorylated human tau at S202/T205 was also faster in anti-aggregant mice and it was similar to that seen in pro-aggregant mice (Fig. 2e, Tables 1 and 2). In order to examine whether this faster turnover is indeed due to faster clearance, we utilized an ELISA that equally detects both murine and human phosphorylated tau using the HJ8.7 antibody (which recognizes both murine and human tau) instead of the human tau specific HJ8.5 antibody. We did not observe faster decline of phosphorylated tau in both pro-aggregant mice and anti-aggregant mice using this assay, suggesting that faster turnover of phosphorylated human tau is likely due to faster clearance not due to faster turnover of the phosphate group itself (Fig. 2e). Both S202/T205 and T231/S235 are located in the proline rich region of tau. In order to examine the effect of phosphorylation in the carboxyl terminal region of tau, we also investigated half-life of human tau phosphorylated at S396. The half-life of tau phosphorylated at S396 in pro-aggregant mice and anti-aggregant mice was 9.8 days and 7.6 days respectively (Fig. 2d, e) which is not as short as the half-life of human tau phosphorylated at the S202/T205 and T231/S235 sites. Taken together, this data suggests that phosphorylation of tau at some sites differentially alters susceptibility of soluble tau to clearance. In fact, it is consistent with the studies showing that some species of phosphorylated tau is preferentially degraded in certain contexts [11, 13].

### Turnover of intracellular insoluble tau in pro-aggregant mice

**Fig. 3:**
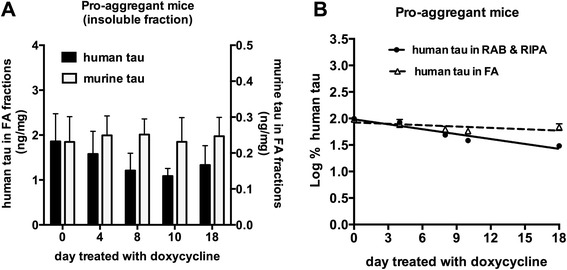
Turnover of intracellular insoluble tau in pro-aggregant mice. **a** Doxycycline specifically reduced human tau in FA fractions of pro-aggregant mice. Human (black bars) and murine tau levels (white bars) in detergent insoluble fractions of pro-aggregant mice following doxycycline treatment were measured. (*n* = 4 for day 10, *n* = 5 forday 4 and day 18, *n* = 7 for day 8, *n* = 12 for day 0.). **b** Insoluble tau showed slower turnover than soluble tau in brain. Semi-log plot of soluble (black circles) and detergent insoluble human (white triangles) tau (Log % human tau) over time in pro-aggregant

The comparison between pro-aggregant mice and anti-aggregant mice revealed that the turnover rate of soluble tau was not different between these mice. Thus we asked whether turnover of tau is altered when it forms insoluble aggregates. Doxycycline resulted in a decrease in human tau in the detergent insoluble fractions without changing murine endogenous tau in pro-aggregant mice although the decrease was not quite statistically significant (Fig. 3a). The estimated turnover rate of insoluble tau was 34.2 days, suggesting that insoluble tau is cleared significantly more slowly than soluble tau (Fig. 3b and Table 1). Tau in insoluble fractions likely represents tau oligomers, fibrils, and other β-sheet enriched assemblies not present in anti-aggregant mice. Thus the data indicates although soluble tau is normally metabolized in pro-aggregant mice, once it forms insoluble structures, it acquires relative resistance to degradation and clearance. Interestingly though, even some clearance of insoluble material occurs suggesting that endogenous mechanisms for aggregate clearance are present.

## Conclusions

In this study, we examined turnover rates of various tau species. We found that in vivo half-life of tau is significantly longer than tau half-life previously reported *in vitro* studies. Therefore, reducing tau production therapeutically will take an appreciable period of time to reduce tau protein levels, especially pre-existing tau aggregates than previously predicted from cell culture studies.

We also asked whether the ΔK280 mutation influences turnover of tau. The half-life of soluble tau was not significantly altered when comparing pro-aggregant to anti-aggregant mice. The data suggests that despite the propensity to altered conformation, the ΔK280 mutation does not lead to a profound effect on clearance of soluble tau. Further studies will be needed to examine whether other tau mutations have an effect on soluble tau clearance.

The mechanistic reason to explain differences in the half-life of various tau species is not known. Furthers studies to understand the mechanisms causing differences in in vivo clearance of tau will be helpful. In addition, the systems that clear extracellular tau in the ISF or CSF will be particularly important to explore since they differ completely from the intracellular degradation pathways important for intracellular tau degradation such as the proteasomal or autophagy systems. Although it was not statistically significant with the number of mice we had, there was a trend that extracellular soluble tau has a longer half-life than intracellular soluble tau in pro-aggregant mice (t_1/2_ = 9.7 day for intracellular soluble tau, t_1/2_ = 17.3 day for extracellular soluble tau, *p* = 0.06, Table 1). This trend was not observed in anti-aggregant mice (t_1/2_ = 11.1 day for intracellular soluble tau, t_1/2_ = 10.9 day for extracellular soluble tau, *p* = 0.44, Table 1) [20]. Although the increasing trend in half-life of extracellular tau in pro-aggregant mice should be further examined with a larger number of mice, one possibility for this trend might be that turnover of extracellular tau is differentially influenced by the presence of tau aggregates, due to an equilibrium between ISF tau and tau aggregates. Such an equilibrium was suggested by our group previously using P301S human tau transgenic mice [21].

Although we are not currently able to detect soluble tau oligomers or aggregates in the ISF by microdialysis, it will be important to understand clearance mechanisms of such extracellular tau species that facilitate the spreading of tau pathology from cell to cell in the brain if present. Besides its role as a diagnostic biomarker in AD, soluble tau in CSF is being evaluated as an endpoint to validate the disease-modifying effects in various clinical trials for AD. In addition to tau released from dying or degenerating neurons, the long half-life of extracellular tau actively released from neurons may provide some important new insights to consider as to what might be leading to elevated extracellular tau as well as tau species that may mediate intercellular tau spreading.

## Methods

### Animals

All animal experiments were performed and approved in accordance with guidelines established by the Animal Studies Committee at Washington University. Regulatable transgenic mice expressing human full-length tau cDNA (Tau 2N4R) with mutations ΔK280/PP (I277P, I308P) (anti-aggregant mice) and mice expressing human full-length tau cDNA (Tau 2N4R) with a ΔK280 mutation (pro-aggregant mice) on C57BL/6 J background of both sexes were utilized. The mice were screened ahead of experiments by quantifying transgene expression via the co-expressed luciferase detected by bioluminescence imaging [6, 15]. P301S transgenic mice (line PS19) which overexpress P301S human T34 isoform tau (1N4R) on a B6C3 background were used to make brain homogenates as a standard for phosphorylated tau measurements [29]. Three month old tau knockout mice were obtained from the Jackson laboratory [30]. Five to nine month old wildtype littermates of P301S transgenic mice were used as wildtype controls. 11.5–12.5 month old hTau mice which express wildtype human tau on a tau knockout background were also used [22].

### in vivo microdialysis

in vivo microdialysis with a 1,000 kDa molecular weight cut-off probe to assess ISF tau levels from awake and freely moving mice was performed as previously described [20].

Briefly, a guide cannula (Eicom microdialysis) was stereotactically implanted in the left hippocampus under isoflurane anesthesia, and cemented. After implantation of the cannula and dummy probes (Eicom microdialysis), mice were habituated to microdialysis cages for one more day. After this recovery period, a 2-mm 1,000-kD cut-off Atmos^LM^ microdialysis probe (Eicom) was inserted through the guide cannula. As a perfusion buffer, 25 % human albumin solution (Gemini Bio Inc.) was diluted to 4 % with artificial CSF (aCSF; 1.3 mM CaCl_2_, 1.2 mM MgSO_4_, 3 mM KCl, 0.4 mM KH_2_PO_4_, 25 mM NaHCO_3_, and 122 mM NaCl, pH 7.35) on the day of use and filtered through a 0.1 μm membrane. A probe was connected to a microdialysis peristaltic pump with two channels (MAB20; SciPro), which was operated in a push-pull mode. Before microdialysis sample collection, a pump was run at the maximum flow rate for at least 1 h and then the flow rate was switched to 0.5 μl/min. To avoid tissue damage, the experimental window was set from 6 to 48 h after probe implantation. ISF samples were collected in a refrigerated fraction collector (SciPro) and analyzed by ELISA.

### Switch off experiments

Sixteen to seventeen month old pro-aggregant or anti-aggregant mice were randomly divided into 5 groups. One cohort of mice was not exposed to doxycycline-containing food pellets (200 mg/kg, Bio-serv) and ISF was collected for 2 days (0 day). Other cohorts of mice received doxycycline-containing food pellets for 4, 8, 10, or 18 days respectively prior to microdialysis. On the 2nd, 6th, 8th, or 16th day, in vivo microdialysis was performed and ISF was collected for additional 2 days. During microdialysis, mice were given doxycycline-containing food pellets. Mean levels of ISF human tau, lactate were plotted from the 3rd, 7th, 9th, or 17th day. On the 4th, 8th, 10th, or 18th day, immediately after the end of microdialysis experiments, brains were collected. Values of % human tau in brain or ISF and % lactate in ISF were normalized by the mean concentration present in the day 0 groups.

### Brain extraction

Mice were transcardially perfused with heparin-PBS and brains dissected and kept at −80 °C until analyzed. Hippocampus was weighed and homogenized in well-defined 3-step serial extraction protocol described previously [16, 21, 28, 29]. Hippocampus was first homogenized with RAB buffer containing (100 mM MES, 1 mM EDTA, 0.5 mM MgSO_4_, 750 mM NaCl, 20 mM NaF, 1 mM Na_3_VO_4_, supplemented by protease inhibitors (Complete, Roche) and phosphatase inhibitors (PhosSTOP, Roche). After centrifugation at 50,000 × g for 20 min, the RAB insoluble pellet was solubilized with RIPA buffer (150 mM NaCl, 50 mM Tris, 0.5 % deoxycholic acid, 1 % Triton X-100, 0.5 % SDS, 25 mM EDTA, pH 8.0, supplemented by protease inhibitor (Complete, Roche) and phosphatase inhibitor (PhosSTOP, Roche)). The RIPA insoluble pellet after centrifugation at 50,000 × g for 20 min was solubilized with 70 % formic acid [21]. Tau levels in these fractions were measured by various tau ELISAs described below. Human tau was detectable in both RAB and RIPA fractions, thus total tau levels in both fractions were used to calculate the half-life of “intracellular soluble tau”. Tau in 70 % formic acid fractions was defined as the “intracellular insoluble tau”. We observed high background values when we measured phosphorylated tau in RIPA fractions with our assays, thus only RAB fractions were used as soluble fractions to reliably measure soluble phosphorylated tau levels. To confirm the specificity of ELISAs used in this study, hippocampus from tau knockout mice, wildtype mice and hTau mice were homogenized with RAB buffer and analyzed.

### Tau ELISAs

Absolute levels of human and murine tau in ISF and brains were measured by ELISAs which use a combination of Tau5/biotinylated HT7 (HT7B) and HJ9.2/biotinylated HJ8.7 (HJ8.7B) monoclonal anti-tau antibodies respectively as previously described [20]. Relative levels of human tau phosphorylated at S202/T205 and T231/S235 were detected by in house ELISAs with a combination of HJ8.5 [28]/biotinylated AT8 (Pierce) and HJ8.5/biotinylated AT180 (Pierce) respectively. To measure total (both murine and human) phosphorylated tau at S202/T205, combinations of HJ8.7/biotinylated AT8 were used. HJ8.5 recognizes human tau specifically whereas HJ8.7 has an identical affinity toward murine and human tau. Human phosphorylated tau at S396 was measured by Tau [pS396] Human ELISA kit (Life technologies). For phosphorylated tau measurement using in house phosphorylated tau ELISAs, RAB soluble fractions from a 7 month old P301S human tau transgenic mouse was used as a standard. Thus, instead of absolute levels, relative levels of phosphorylated tau normalized to “0 day group” were used for half-life calculation.

### Lactate measurements in ISF

ISF lactate levels were determined with a YSI2700 biochemistry analyzer (YSI Life Sciences), which quantifies their levels with immobilized enzyme biosensors specific for the substrates [20].

### The calculation of half-life

The half-life of tau was calculated as we previously reported using the slope of linear regression that includes all data points (t_1/2_ = 0.693/k, where *k* = 2.303 k’) (Fig. 1c) [20, 23]. In order to consider the time required for transgene switch off in our strategy, we measured luciferase activity [6]. Following doxycycline treatment, bioluminescence signal intensity by luciferase was constant for 4 h and then started to decline (unpublished observation of KH and EMM). This 4 h-delay time consists of less than 5.3 % of the half-life of any form of tau assessed in this study, suggesting that the contribution of this delay to half-life calculation is negligible.

### Statistical analysis

Data in figures represent mean ± s.e.m. The comparison of multiple groups was done by one-way ANOVA with Tukey’s post hoc test. Correlation coefficients (R^2^) were calculated to see the goodness of fit for the linear regression. To examine statistically significant differences in turnover rates, the slope of linear regression that includes all data points over time were compared using analysis of covariance (ANCOVA) [31].

## Abbreviations

AD: Alzheimer’s disease
CSF: Cerebrospinal fluid
NFTs: Neurofibrillary tangles
HJ8.7B: (Biotinylated HJ8.7)
HT7B: (Biotinylated HT7)
ISF: Brain interstitial fluid
RAB: Reassembly buffer
